# Supplementary material for: Patterns of Intron Gain and Loss in Fungi
Source: PLoS Biol. 2004 Nov 30;2(12):e422. doi: 10.1371/journal.pbio.0020422 (PMC532390; doi:10.1371/journal.pbio.0020422)
Supplement: Table S1 — Also available at http://genes.mit.edu/NielsenEtAl/. (4.3 MB ZIP). [file pbio.0020422.st001.zip › NielsenEtAl/html/88.html]

AN8169.1.NCU03768.1.MG04017.1.FG07410.1


```
 CLUSTAL W (1.82) Multiple Sequence Alignments - Introns Inserted


Sequence 1: NCU03768.1	595 aa
Sequence 2: MG04017.1	540 aa
Sequence 3: FG07410.1	532 aa
Sequence 4: AN8169.1	543 aa
Alignment Length: 605 aa
Number Identitical Residues: 221 aa
Alignment Score (without introns) 11072


MG04017.1 	----MGSPTITPGQTTPSSYPESRVLIIITGGTICMQPSDDGLVPVDSFVERAMAPLPSF
NCU03768.1	----MGQVSFE-GKNPESHYPESRVLIIMTGGTICMQPSADGLVPMTGFLKNAMAPRPSF
FG07410.1 	-MSRAGSPGLPVKVVPETSYPESRVLIIGTGGTICMQQGPDGLQPTDNFLENAMAPRSSF
AN8169.1  	MVAADDTIPELAEGHMEPPVPESRVLIIMTGGTICMRPSPAGLVPARDFQKQCLARVPTF
          	  :  .           .  ******** *******: .  ** *  .* :..:*  .:*

MG04017.1 	ND-ASPG~T~E1-LHAYHHGKELLLPSLRTPPSSYSRHIRYCALEFSPLLDSSSISSAGW
NCU03768.1	NDPTAPN~V1Q~-LRAYKNGAKLTLDSLRTPVSAYSRHIRYGILEFTPLLDSSSISSMGW
FG07410.1 	NDFSNPG1T~-~-LPAVRDGAKIQIGSLRTPATAYNRHVRYGIIEFSPLLDSSSISAHDW
AN8169.1  	NDGSNST~M~M~DVVTNAVGEIKAHVSLRTPLTAYGRRVRYTVFEFDELLDSSSIDAKGW
          	** : .      .: :   *      ***** ::*.*::**  :**  *******.: .*

MG04017.1 	AEIAQTVKENYHLFDGFVVLHGTDSLAYSASALSFMMADLGKSVILTGSQASIFALQTDA
NCU03768.1	TEIALTIKENYHMFDGFVVLHGTDSLAYTASALSFMMSDLGKPVILTGSQASIFALQSDA
FG07410.1 	DAMASCVEENYHLFDGFVILHGTDSLAYTASALSFMMENLGKPVILTGSQAPIFALQSDG
AN8169.1  	SEIARTIERNYTLFDGFVVLHGTDSLAYTCSALSFMLQNLGKPVILTGAQAPMLELQNDA
          	  :*  ::.** :*****:*********:.******: :***.*****:**.:: **.*.

MG04017.1 	VDNLLGSLIIAGTFVVPE~VCLFFHHALYRGNRTTKVSASAFAAFDSPNCPPLAKVTSLG
NCU03768.1	VDNLLGSLIIAGTFVIPE~VCLFFHHTLYRGNRTTKVSASSFEAFDSPNCDPLAKVTSLG
FG07410.1 	VDNLLGSLIIAGTFTIPE~VCLFFHHRLYRGNRSAKVSATEFEAFASPNSEPIAKVNGLG
AN8169.1  	TDNLLGSLVVAGHFMIPE0V-----------------AASDFNAFNSPNCAPLAVTTSMR
          	.*******::** * :** *                 :*: * ** ***. *:* ...: 

MG04017.1 	VDVNWQLVRRPTRIAGFQVTTYLDTAHVACLRIFPGIKPEMLDSVLRVPGLKGLILETFG
NCU03768.1	VDVNWALIRRPTQIAEFQVTKYLDTAHVACLRIFPGIKPEMIESVLKVPDLRGLILETFG
FG07410.1 	ISVNWPLVLRPTSIAKFHVTKGLDTTHVACLRVFPGIKPEMIDAVLHLPDIRGLILETFG
AN8169.1  	TNVNWEIVNRPNSIEHFSIQTNLDTTHVACLRIFPGIKPEMVDAVLKLEGLRGLVLETFG
          	 .*** :: **. *  * : . ***:******:********:::**:: .::**:*****

MG04017.1 	MGNAPSGVDGSLTKVIRAAVEREIVIVNVSQC~TNGFVSPLYAPGTALGRAGVVFGQDLT
NCU03768.1	MGNAPGGVDGSLTKVIKEAIDRGTVIVNVSQC~TNGFVSPLYAPGTVLGRAGVVFGHDLT
FG07410.1 	MGNIPGGSDGPLTRIIKDAVERGIVVVNVSQC~VNGFVSPVYAPGTVLGRAGVIFGLDLT
AN8169.1  	AGNAPHGQDNAMTKVLADAIARGIVIVNVTQC1LTGSVSPVYAPGMSLSKAGVVAGLDMT
          	 ** * * *..:*:::  *: *  *:***:**  .* ***:****  *.:***: * *:*

MG04017.1 	TEGALTKLSYLLAIEGLSYADITSRMAQSLRGEMTETSVPSFSHPAGSVDYGGVASGNLT
NCU03768.1	TEAALTKLSYLLALPNLTYSEITARMSQSLRGEMTEMATPSFSHPAGSIDS---AMAWLP
FG07410.1 	AEAALAKVSYLLAQPDLSTKEIQEKLSRSLRGEMTEIAHQSFSNPAGSLDF---AAAHLT
AN8169.1  	TEAALTKLAYLLALPGADPKLVASKMSVSLRGELTESSQPVFRHPDG----------ALP
          	:*.**:*::****  .     :  ::: *****:** :   * :* *           *.

MG04017.1 	AVE~TRFTELGYAIRNGDLEAVRRMLQGDEHSTHPLLKTSDYAGNTAV~HLAAVGPKAEV
NCU03768.1	AAD1TAFTALGYAIRNGDLRTVREILEGDEFN-HQLLKRADYVGNTPV~HLAAVGPNPDV
FG07410.1 	ASE~RAFSALGYAIENGELKLVKELLQG---EGAQLLKQADYAGNTAV~HLAAVAGKTDI
AN8169.1  	ERV~QALTILGYAIAQGDLARVEELLKS---EHHWLLNDADYSGNTPM0HLAATSPSVDI
          	      :: ***** :*:*  *..:*:.   .   **: :** ***.: ****.. . ::

MG04017.1 	LREVLTRGGSVHLRNRGNNTALFLAEKMGNEECVKLLREAGAHLWETE------------
NCU03768.1	LHELLIRGASVHPRNYANHTPLYLAEKMGNVENVKLLKEAGAHLWRTEPHSGNVSEAGDL
FG07410.1 	LLELLRLGASVHERNKANLSPLYVAVKSGQEPCAQLLRIAGAHLAVEE------------
AN8169.1  	LRFLLLQGGSVHLRNRNGRTPLFLAANAGLSEHVLLLRKSGAHLHSDERTAAQLLARRRP
          	*  :*  *.*** **  . :.*::* : *    . **: :****   *  :..       

MG04017.1 	------------------------RLHQHKQQT--ETGEDEQPRK---RAHVAS------
NCU03768.1	ASTTDVASVGAADDDKEVKDVSEHRSNEKEQKTNDQVADTVSPRHGLDKSHLVTDESLVH
FG07410.1 	--------------------------RET-------MPGYVTPKY----SHFGKHEL---
AN8169.1  	GIWG-------------------LAGIGPREISDREMEEVDGDGYRAGSSHWPMAGSAP-
          	.                             . :. .           . :*         

MG04017.1 	------------
NCU03768.1	GLKKEDIGKFVN
FG07410.1 	------------
AN8169.1  	------------
          	
```
